# Estrous cycle influences excitatory amino acid transport and visceral pain sensitivity in the rat: effects of early-life stress

**DOI:** 10.1186/s13293-016-0086-6

**Published:** 2016-07-14

**Authors:** Rachel D. Moloney, Jahangir Sajjad, Tara Foley, Valeria D. Felice, Timothy G. Dinan, John F. Cryan, Siobhain M. O’Mahony

**Affiliations:** Laboratory of Neurogastroenterology, APC Microbiome Institute, University College Cork, Cork, Ireland; Department of Anatomy and Neuroscience, University College Cork, Office 4.113, Western Gateway Building, Cork, Ireland; Department of Psychiatry and Neurobehavioural Science, University College Cork, Cork, Ireland; Present Address: Oklahoma Center for Neuroscience, University of Oklahoma Health Science Center, Oklahoma City, OK USA

**Keywords:** Visceral pain, Colorectal distension, Early-life stress, Excitatory amino acid transporter, Glutamatergic system, Aspartate uptake

## Abstract

**Background:**

Early-life stress (ELS) is a recognized risk factor for chronic pain disorders, and females appear to be more sensitive to the negative effects of stress. Moreover, estrous cycle-related fluctuations in estrogen levels have been linked with alternating pain sensitivity. Aberrant central circuitry involving both the anterior cingulate cortex (ACC) and the lumbosacral spinal cord has also been implicated in the modulation of visceral pain in clinical and preclinical studies. Here we further investigate changes in visceral pain sensitivity and central glutamatergic systems in rats with respect to estrous cycle and ELS.

**Methods:**

We investigated visceral sensitivity in adult female Sprague-Dawley rats, which had undergone maternal separation (MS) in early life or remained non-separated (NS), by performing colorectal distension (CRD). We also assessed excitatory amino acid uptake through excitatory amino acid transporters (EAATs) in the lumbosacral spinal cord and ACC.

**Results:**

NS animals in proestrus and estrus exhibited reduced EAAT uptake and decreased threshold to CRD. Moreover, total pain behaviors were increased in these stages. MS rats exhibited lower pain thresholds and higher total pain behaviors to CRD across all stages of the estrous cycle. Interestingly, cortical EAAT function in MS rats was inhibited in the low estrogen state—an effect completely opposite to that seen in NS rats.

**Conclusions:**

This data confirms that estrous cycle and ELS are significant factors in visceral sensitivity and fluctuations in EAAT function may be a perpetuating factor mediating central sensitization.

## Background

Chronic pain syndromes such as irritable bowel syndrome (IBS), fibromyalgia, migraine, and interstitial cystitis display a striking female preponderance with females presenting at the clinic up to ten times more often than their male counterparts [1, 2]. Moreover, females report more intense pain, of longer duration, and more frequently. With these high prevalence rates, a growing body of evidence suggests that gonadal hormones play a significant role in pain processing [3]. In line with this, recent evidence suggests the possible interaction of gonadal hormones with the brain-gut axis and downstream pain processing [4–6]. Indeed, gonadal hormone binding sites are widely distributed in areas of the central nervous system (CNS) involved in pain processing. Sex steroids also have receptors located throughout the entire intestine, with both estrogen and progesterone exhibiting direct effects on visceral organs [7]. Furthermore, estrogen has been implicated in the possible modulation of visceral pain perception [5, 8]. However, both clinical studies and animal models have revealed conflicting evidence on the role of the female hormonal cycle on pain perception [9, 10]. Numerous studies in the rat have shown a decreased threshold and greater sensitivity in the proestrus phase; however, others have reported no difference between estrous stages [5, 11–13]. These discrepancies may be due in part to strain differences and the inaccuracy of estrous cycle determination.

Estrogen receptors have also been shown to interact with other key neurotransmitter systems such as the glutamatergic system, which itself is critical to pain processing mechanisms [14]. In particular, the process of central sensitization implicates a critical role of excessive glutamatergic signaling leading to the development of plasticity, thus maintaining the chronic pain state [15, 16]. The glial excitatory amino acid transporters (EAATs), in particular, EAAT 1 and EAAT 2 are crucial in the maintenance of homeostasis within the glutamatergic synapse; however, their expression has been shown to be altered in chronic pain models [17, 18]. Intrathecal administration of EAAT blockers in naïve animals results in spontaneous somatic pain, as well as hyperalgesia and allodynia, implying that a continuous spinal glutamate uptake has a key basal anti-nociceptive action [19–21]. Moreover, EAAT 2 has also been shown to play a vital role in numerous models of visceral pain [22] with increased expression showing anti-nociceptive effects [23]. Moreover, pharmacological activation of these transporters has also demonstrated therapeutic potential [17].

Many chronic pain disorders are also included in categories of stress-induced disorders as a significant proportion of patients attribute the onset and exacerbation of their symptoms to an early-life stressful period or chronic stress throughout life. Indeed, it is now becoming more evident that females appear more sensitive to the negative effects of stress [24]. Early-life stress (ELS) during childhood is related to increased risk to develop depression, anxiety, and chronic pain in adulthood [25]. Maternal separation in the early days of life in animals is a widely used model of ELS and has been used to elucidate the underlying mechanisms of depression as well as chronic pain disorders [26–28]. The model is based on the evidence that adverse environmental alterations during early life can cause long-lasting effects into adulthood e.g., increased visceral pain sensitivity [27]. In the present study, we aimed to assess whether maternal separation and the estrous cycle alter visceral sensitivity and if changes in phenotype were associated with changes in excitatory amino acid transport via glial uptake in the lumbosacral spinal cord and the anterior cingulate cortex (ACC), two critical regions within the CNS known to play a role in visceral pain processing [26, 29].

## Methods

### Animals

Adult male and female Sprague-Dawley rats (250–300 g) (Harlan, UK) were used as breeding partners to generate offspring in this study. Upon arrival, animals were housed according to sex, four to five animals per cage, in plastic cages and were maintained in a temperature-controlled room (20 ± 1 °C) with a 12-h light/dark cycle (7:00 am to 7:00 pm). The animals were allowed 1 week to acclimatize to the animal facility in University College Cork after arrival. Breeding pairs were housed together until confirmation of pregnancy. Females were then group housed throughout gestation until gestation day 19 after which time they were housed singly and allowed to give birth. Two cohorts of animals were used in the current study. (1) The first cohort was used for behavioral analysis of visceral sensitivity. (2) The second cohort was used for naïve sample collection to assess EAAT function. Group sizes were nine to ten animals and was based on previous experiments. All experiments were conducted in accordance with the European Directive 2010/63/EU and approved by the Animal Experimentation Ethics Committee of University College of Cork.

### Maternal separation

Maternal separation was performed from postnatal day 2 (PND 2) to PND 12 inclusive as previously described [28, 30]. Briefly, litters were randomly assigned to either the non-separated (NS) group or the maternally separated (MS) group. NS animals were left undisturbed except for routine husbandry practices. MS pups were separated daily from their mothers in a separate room and placed in a clean cage with fresh bedding, placed on top of heated pads (30–33 °C) for 3 h from 9:00 am until 12:00 pm after which time animals were placed back in their mother’s home cage. Following the separation period (PND2–PND12), animals were left undisturbed except for weekly cage cleaning. Offspring were weaned and sexed at PND 21. Animals were allowed to mature to adulthood (8 weeks), and all female animals were used for the remainder of the study.

### Vaginal smears

Females were vaginally lavaged daily with saline for at least two consecutive estrous cycles, and cells were immediately viewed under a microscope prior to behavioral assessment. Those rats that were regularly cycling were used in this study. The stage of estrous cycle was determined as previously described [13]. Since metestrus only lasts for a short period (5–6 h) and the plasma estrogen concentration in metestrus do not differ from that in diestrus, data from these two groups of rats were pooled. For cohort 1, the behavioral study, animals were lavaged immediately before balloon insertion into the colorectum, to assess estrous stage during visceral pain measurement. For cohort 2, tissue collection only, the animals underwent vaginal smears for cycle phase estimation immediately before decapitated for tissue collection. Towards the end of each study, there were occasions where the lavaged rats were not in the correct phase (we aimed for nine to ten per group) and then we waited a day or two until we lavaged again to check the stage and if it fit into the groups that required more numbers.

### Colorectal distension

Colorectal distension (CRD) was performed as previously described [31, 32]. Briefly, animals were fasted overnight and anesthetized with isoflurane (3–5 % in oxygen) followed by insertion of a 6-cm latex balloon into the colorectal cavity, 1 cm from the anus. The animals were allowed to recover for 10 min before CRD commenced (9:00 am to 12:00 pm) in unrestrained freely moving animals. The paradigm used was an ascending phasic distension from 0 to 80 mmHg over 8 min. The parameters of interest were (1) the threshold pressure (mmHg) that evokes the first visually identifiable visceral pain behavior and (2) the total number of pain behaviors over the distension period. Postures defined as visceral pain behaviors were abdominal retractions and/or abdominal withdrawal reflex. The experimental groups were randomized, and behavioral testing was performed by an experimenter blinded to treatment groups to eliminate any bias. All animals underwent CRD only once.

### Sample preparation for aspartate transport assay

The animals were euthanized by decapitation immediately after vaginal smearing. Their spinal cords were removed by hydraulic pressure into Hank’s balanced salt solution (HBSS)-filled Petri dishes, and 0.4-mm thick slices were obtained from lumbosacral spinal cord using a McIIwain tissue chopper. Similarly, brain tissue was removed from the skull and sectioned using a vibratome to obtain ACC sections. These slices were separated by fine dissection under a microscope and transferred to 24-well culture plates filled with both sodium containing HBSS (labeled as Na^+^) and sodium-free HBSS (labeled as Na^−^) separately. Na^+^ plate was maintained at 35 °C and Na^−^ on the ice. The slices from Na^+^ plate were washed once with l mL of 35 °C HBSS and Na^−^ plate with l mL of 4 °C sodium-free HBSS to assess sodium-dependent and independent uptake, respectively.

### Aspartate transport assay

Since EAATs show high affinity to both glutamate and aspartate, we used aspartate. This protocol has been previously described in brain slices [33]; we optimized this technique to be used on spinal cord slices for the first time. Aspartic acid, d-[2,3-^3^H] (specific activity 12.9 Ci/mmol), was purchased from PerkinElmer, USA. RIPA buffer and Pierce BCA protein assay kit were purchased from Fisher Scientific Ireland. All other reagents were purchased from Sigma-Aldrich. HBSS was prepared containing (in mM): 137 NaCl; 0.63 Na_2_HPO_4_; 4.17 NaHCO_3_; 5.36 KCl; 0.44 KH_2_PO_4_; 1.26 CaCl_2_; 0.41 MgSO_4_; 0.49 MgCl_2_ and 1.11 glucose, in pH 7.2. In sodium-free HBSS, NaCl was replaced by 137 mM *N*-methyl-d-glucamine.

Spinal cord and ACC slices were pre-incubated at 35 °C and ice in Na^+^ and Na^−^ HBSS, respectively, for 30 min. Then 0.66 μCi/ml aspartic acid, d-[2,3-^3^H], and 100 μM (final concentration) in 20 μl cold d-aspartate was added. After 3 and 7 min of incubation of spinal cord and ACC, respectively, the slices were washed twice with 1 ml of corresponding ice-cold HBSS. Tissue was transferred into 1.5-ml tubes containing RIPA buffer and was mechanically dissociated with pestles. This mixture was homogenized for 15 min at 4 °C and the residue was removed. Radioactivity was measured in terms of counts per minute (CPM) using a liquid scintillation counter and results for Na^−^ samples were subtracted from those of Na^+^ samples to achieve sodium-dependent uptake-hallmark of EAAT 1 and 2 function. Uptake procedure was performed in triplicate. Protein was measured using Peirce BCA protein assay kit. Final scintillation result for each slice was divided by respective protein value to achieve aspartate uptake in terms of CPM/mg-indicative of spinal and cortical EAAT’s function.

### Statistical analysis

All data was normally distributed according to Gaussian distribution analysis. Data are expressed as mean ± SEM. Two-way analysis of variance (ANOVA) and Tukey post hoc test were used in all cases. *p* < 0.05 were considered statistically significant. The sample size (*n* = 9/10) was based on previous studies showing that it was sufficient to observe statistically significant results.

## Results

### Early-life stress and estrous cycle-dependent variations in visceral sensitivity

**Fig. 1:**
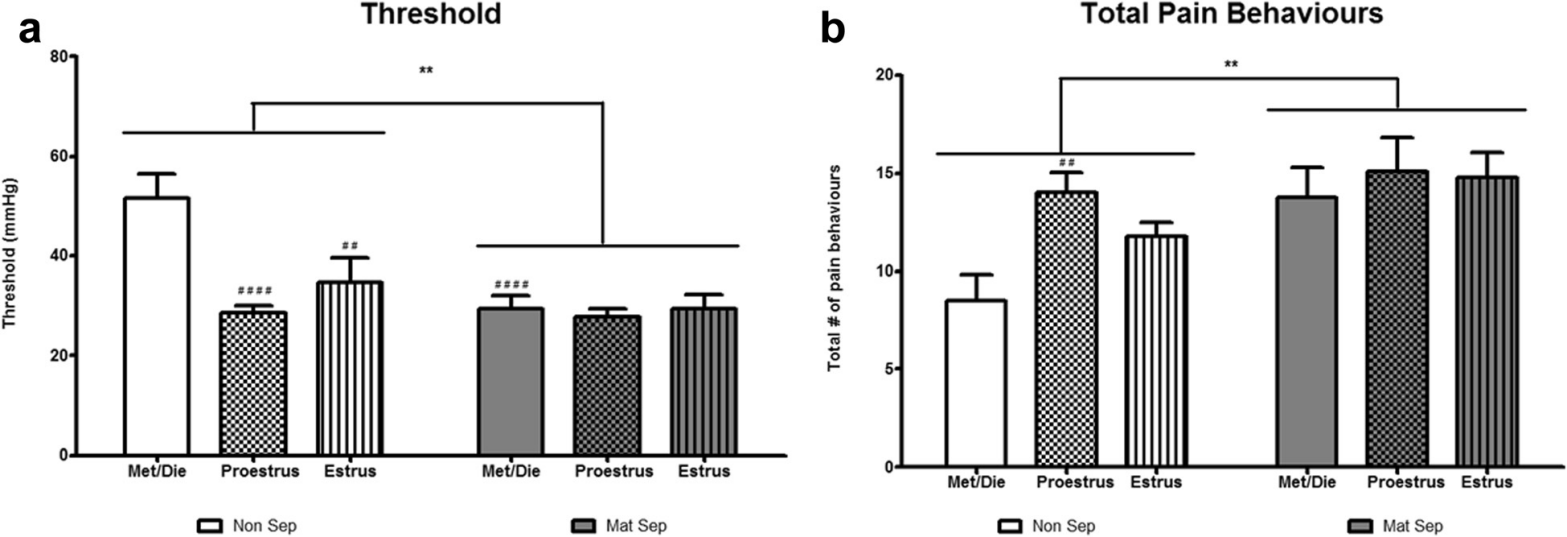
Early-life stress and estrous cycle-dependent variations in visceral sensitivity. Maternally separated (MS) animals exhibit visceral hypersensitivity with a significantly lower threshold of visceral distension required for identifiable abdominal contraction (**a**) and increased total pain behaviors (**b**) compared to non-separated controls (NS) (**p < 0.01 non-sep vs mat-sep, ^##^ *p* < 0.01, ^####^ *p* < 0.0001 vs met/die non-sep, *n* = 9–10/group)

A two-way ANOVA analysis of threshold sensitivity revealed a significant effect of stress (*F*_(1, 50)_ = 11.42, *p* < 0.01), estrous cycle (*F*_(2, 50)_ = 6.847, *p* < 0.01), and an interaction effect of stress × estrous cycle (*F*_(2, 50)_ = 5.561, *p* < 0.01, Fig. 1a, *n* = 9/10 per group). Overall, MS rats displayed a lower threshold compared to NS rats. Post hoc analysis revealed significant differences between groups with NS animals in both the proestrus (*p* < 0.0001) and estrus (*p* < 0.01) phases exhibiting decreased threshold values compared to NS metestrus/diestrus animals. In addition, a difference was noted between MS rats and NS rats in metestrus/diestrus (*p* < 0.0001). There were no between-group differences observed in MS animals.

A two-way ANOVA analysis of total pain behaviors also revealed a significant effect of stress (*F*_(1, 50)_ = 8.657, *p* < 0.01) and a significant effect of estrous cycle (*F*_(2, 50)_ = 3.571, *p* < 0.05) but no interaction effect of estrous cycle × stress (*F*_(2, 50)_ = 1.305, *p* > 0.05, Fig. 1b, *n* = 9/10 per group). Overall, MS rats showed a higher number of pain behaviors, with post hoc analysis revealing significantly increased total pain behaviors in the NS animals in the proestrus (*p* < 0.01) phase compared to NS animals in the metestrus/diestrus phase. There were no between-group differences observed in MS animals.

### Early-life stress and estrous cycle-dependent variations in central EAAT activity

#### Lumbosacral spinal cord

**Fig. 2:**
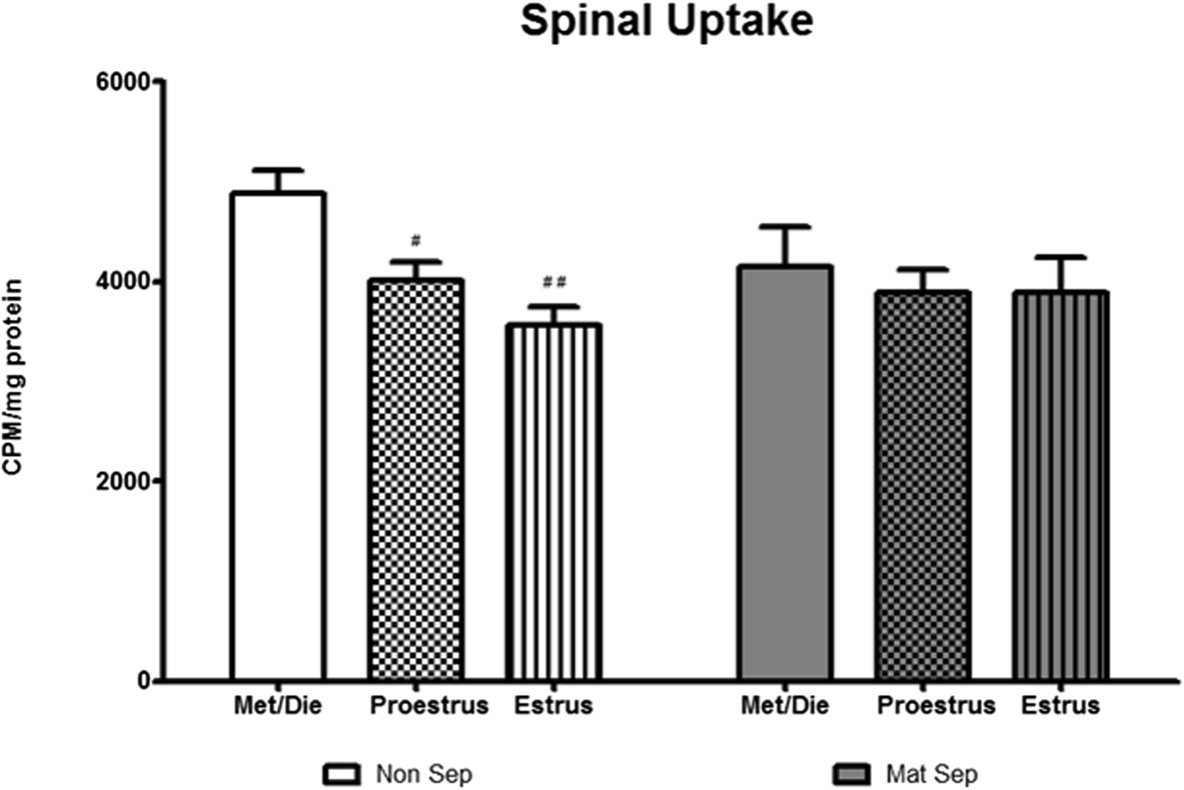
Early-life stress and estrous cycle-dependent variations in spinal EAAT function. Estrous cycle plays a role in EAAT function in the lumbosacral spinal cord with both the estrus and proestrus phases of the cycle inducing significant reductions in EAAT function (^#^ *p* < 0.05, ^##^ *p* < 0.01 vs met/die non-sep, *n* = 10/group)

Two-way ANOVA analysis of EAAT function within the lumbosacral spinal cord revealed a significant effect of the estrous cycle (*F*_(2, 53)_ = 4.349, *p* < 0.05, Fig. 2, *n* = 10 per group) but no significant effect of stress or interaction effect. Post hoc analysis revealed NS animals in the estrus phase (*p* < 0.01) and the proestrus phase (*p* < 0.05) to have reduced EAAT function compared to NS animals in the metestrus/diestrus phase.

#### Anterior cingulate cortex

**Fig. 3:**
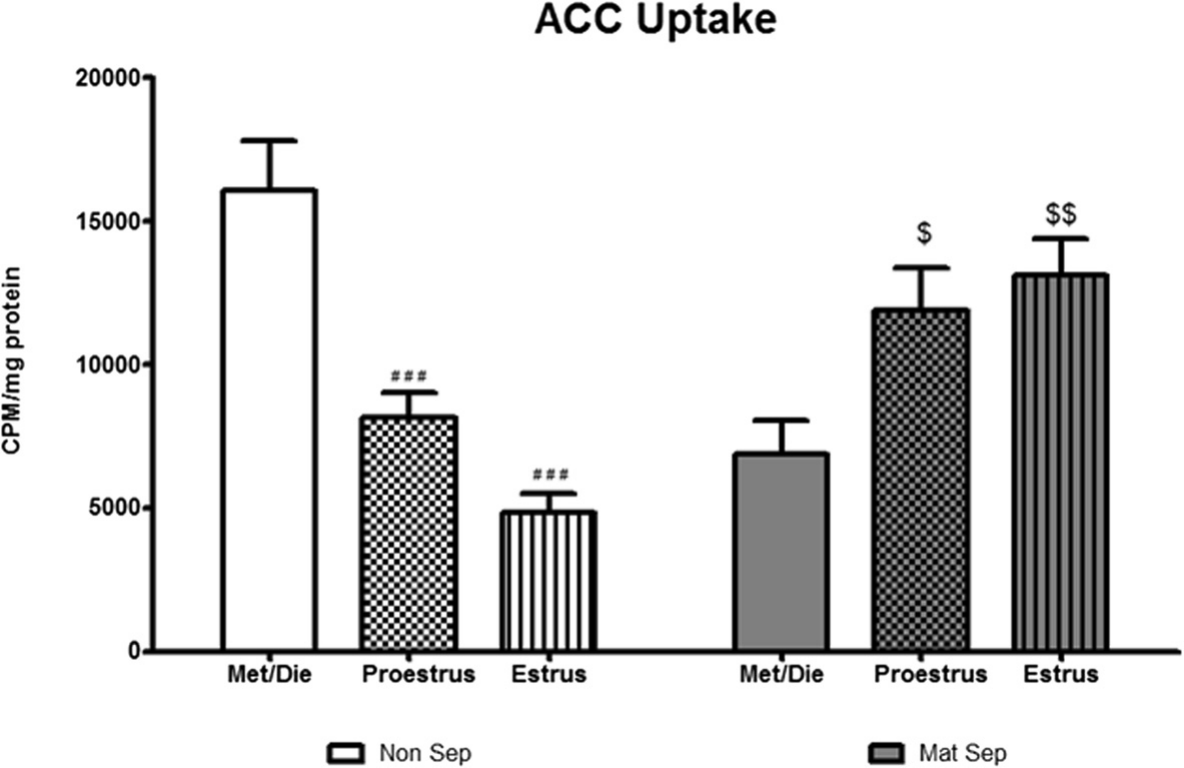
Early-life stress and estrous cycle-dependent variations in central EAAT function. Estrous cycle and early-life stress play a role in EAAT function in the anterior cingulate cortex with both the estrus and proestrus phases of the cycle inducing significant reductions in EAAT function in NS rats (^####^ *p* < 0.0001 vs met/die non-sep, *n* = 10/group) and estrus and proestrus phases showing increased EAAT function in MS rats (^$^ *p* < 0.05, ^$$^ *p* < 0.01 vs met/die mat-sep, *n* = 10/group)

Moreover, a significant interaction of stress × estrous cycle was observed for EAAT function within the ACC (*F*_(2, 51)_ = 27.00, *p* < 0.0001, Fig. 3, *n* = 10 per group) with post hoc analysis revealing significant decreases in both the proestrus (*p* < 0.0001) and estrus (*p* < 0.0001) phases in NS animals. However, cortical EAAT function in MS rats was inhibited in low estrogen state i.e., diestrus, rather than high estrogen states—an effect completely opposite to that seen in NS rats.

## Discussion

Here we show that ELS and the estrous cycle play significant roles in visceral sensation. MS animals displaying similar visceral pain responses at all stages of the cycle while in NS controls, fluctuations in visceral sensitivity occurred across the cycle. We noted an early-life stress-induced increase in visceral sensitivity in the combined metestrus and diestrus phases but not in the proestrus and estrus phases.

Furthermore, excitatory amino acid transport, both within the lumbosacral spinal cord and the ACC, were also altered in response to stress and estrous cycle, thus indicating that aberrant excitatory transport may in part lead to fluctuations in visceral sensitivity.

Functional gastrointestinal disorders, characterized by visceral pain, have been more commonly reported in females, in particular, premenopausal women, with increasing focus on the effect of hormonal cycles on pain processing [34]. Female IBS patients report increased visceral pain during menses suggesting enhanced visceral sensitivity during the perimenstrual period [9]. The hormonal cycle in the rodent is much shorter than that in humans with the cycle completed in just 4–5 days. Progressing through the stages may occur over the course of just a few hours, making it difficult to investigate the role of the estrous cycle in nociceptive assays due to the inconsistent methodology. For this reason, many studies disregard the cycle [35–37]; however, it is increasingly more appreciated that this is a significant confound and makes the interpretation of data difficult. Indeed, the estrous stage has previously been shown to alter the responses in somatic nociceptive assays, in particular, the tail flick test [10, 38, 39]. This current study found significant changes in visceral sensitivity across the stages of estrous cycle in controls, with heightened visceral pain response in the proestrus and estrous phases. This is in agreement with previous reports [12, 13]; however, it is pertinent to note that other studies have shown findings on the contrary, with evidence showing no changes in visceral pain throughout the cycle [11]. These discrepancies seen in animal models may be due in part not only to species and strain differences but also to the impact of stress. Although not widely acknowledged in the literature, the methodology used to assess estrous stage, i.e., vaginal lavage, can be stressful to animals and may result in changes in the cycle. Thus, it is important to acclimate animals to the vaginal smearing process itself as was performed in this study.

The most potent and prevalent endogenous estrogen, estradiol, is at its highest level during the proestrus phase, which also coincides with the highest level of visceral pain sensitivity, highlighting the potential role of the gonadal hormones in visceral pain. Moreover, its contribution to visceral hypersensitivity has been investigated in animal models through ovariectomy and hormone replacement studies [5, 6]. These studies reported reduced visceral sensitivity in ovariectomized females compared with intact females. Estrogen replacement was reported to increase visceral sensitivity back to levels of that seen in the intact females [5]. Furthermore, the implantation of micropellets containing estradiol and progesterone into the amygdala increased visceral pain behaviors in ovariectomized rats, with no differences seen when placed in neighboring brain regions [6]. Interestingly, no changes were observed in somatic pain thresholds. These findings suggest that female sex steroids do indeed play a significant role in visceral nociception.

Stress and altered hypothalamic-pituitary-adrenal (HPA) axis function is a significant risk factor for the development of visceral pain. Thus, it is interesting to note that hormonal fluctuations have also been shown in stress responsivity and altered neurobiology of the HPA axis. Females have been found to have higher corticotropin-releasing hormone, adrenocorticotropic hormone, and corticosterone levels during proestrus, the phase of the cycle in which estradiol levels are higher than during other phases of the estrous cycle [40–42]. Furthermore, plasma adrenocorticotrophin hormone and corticosterone levels in response to stress are higher during proestrus than during other phases of the cycle [43–45]. Moreover, neurotransmitter systems implicated in the control of HPA function [46, 47] show variations related to the estrous cycle and are sensitive to gonadal steroid levels [48–50]. Indeed, here we specifically focus on the glutamatergic system and the role of glial glutamate transport in visceral nociception.

Here we show that the activity of glutamate transporters in the lumbosacral spinal cord and ACC are significantly decreased during the phases of the estrous cycle when visceral sensitivity is higher. The EAATs are responsible for removing excess glutamate from the synaptic cleft thereby reducing the availability of glutamate and preventing central sensitization. Studies from our own group have shown expression of these transporters is altered in animal models of heightened sensitivity to colorectal distension including the maternal separation model [17] as well the CBA/J strain of mouse [18].

Water avoidance stress have also been shown to alter expression levels of these transporters in the spinal cord which was positively correlated with pain behaviors [22, 51]. Taken together, it is plausible that glutamate uptake enhancers may prove to be a novel therapeutic strategy for the treatment of visceral hypersensitivity [17]. Indeed, recent studies show down-regulation of spinal EAAT transporters in various models of chronic pain [52–54]. Moreover, the importance of glial glutamate uptake is further highlighted by reports where EAAT overexpression reduces pain sensitivity [23, 51, 55]. Previous studies have shown higher glutamate concentration in ACC in animals exposed to ELS [56].

Overall, in MS rats, stress increased visceral sensitivity in metestrus/diestrus phases but had no effect in proestrus and estrus compared to NS rats. The reason for this is not clear, but it is interesting to note that glutamate uptake in the spinal cord paralleled this. However, uptake in the ACC was opposite to the that of the NS controls. This may be compensatory as uptake was higher in the proestrus and estrus in the MS animals, which may indicate that higher activity of these transporters is necessary to maintain behaviors at the same level. Moreover, it appears that visceral sensitivity in MS rats is linked to changes in EAAT function in the ACC, while both the spinal and ACC EAAT function was related to pain behaviors in the NS group. Both ACC and spinal cord EAAT function are reduced in the higher estrogen state i.e., proestrus and ensuing estrus, paralleling that of the pain behaviors. Other studies have indicated that estrogen can affect the expression of the EAATs as well as l-glutamate uptake activity in cultured midbrain astrocytes [57]. Estrogen exerts if effects through either membrane bound or nuclear receptors and stimulation of PI3-kinase coupled to nitric oxide production may be involved in the inhibitory regulation of glutamate transporter activity by estrogen. The presence of nuclear estrogen receptors in astrocytes has been demonstrated in vitro and in vivo [58]. Moreover, it should be appreciated that the expression of estrogen receptors can change [58] and hence influence the level of impact of estrogen changes also.

Our data further supports an important role of glutamate transport at both the level of the spinal cord and higher brain centers in the pathophysiology of visceral pain.

## Conclusions

The findings presented here highlight the important contribution of female sex hormones to the control, processing, and manifestation of visceral pain. Indeed, our studies suggest a complex link between steroid signaling, stress, and glutamatergic neurotransmission. Taken together, these findings have added to the accumulating literature implicating the role of sex hormones and glutamate in visceral pain.

## Abbreviations

ACC, anterior cingulate cortex; ANOVA, analysis of variance; CRD, colorectal distension; EAAT, excitatory amino acid transporter; ELS, early-life stress; HBSS, Hanks balanced salt solution.; HPA, hypothalamic-pituitary-adrenal axis; IBS, irritable bowel syndrome; MS, maternal separation; NS, non-separated; PND, postnatal day
